# Risk Factors for COVID-19 Associated Mucormycosis: The Ophthalmologist’s Perspective

**DOI:** 10.3390/jof8030271

**Published:** 2022-03-08

**Authors:** Alper Bilgic, Laurent Kodjikian, Aditya Sudhalkar, Shyamal Dwivedi, Viraj Vasavada, Arpan Shah, Mikhail Dziadzko, Thibaud Mathis

**Affiliations:** 1 Alphavision Augenarztpraxis, 27568 Bremerhaven, Germany; drbilgicalper@yahoo.com (A.B.); adityasudhalkar@icloud.com (A.S.); 2 Service d’Ophtalmologie, Centre Hospitalier Universitaire de la Croix-Rousse, Hospices Civils de Lyon, Université Claude Bernard Lyon 1, 69004 Lyon, France; laurent.kodjikian@chu-lyon.fr (L.K.); mikhail.dziadzko@chu-lyon.fr (M.D.); 3 UCBL, INSA Lyon, CNRS, MATEIS UMR5510, Université de Lyon, 69100 Villeurbanne, France; 4 MS Sudhalkar Medical Research Foundation, Baroda 390001, India; shyamal@raghudeepeyeclinic.com (S.D.); viraj@raghudeepeyeclinic.com (V.V.); 5 Pranayam Heart and Lung Institute, Baroda 390001, India; iseemadison@gmail.com; 6 INSERM, U1290 RESHAPE, Université Claude Bernard Lyon 1, 69004 Lyon, France

**Keywords:** COVID-19, fungus, immunosuppression, mucormycosis, SARS-CoV-2

## Abstract

The COVID-19 pandemic has led to a dramatic rise in the incidence of rhino-orbito-cerebral mucormycosis (ROCM) in India. The purpose of our report is to describe the prevalence of ROCM in the context of SARS-CoV-2 infection during the second Indian COVID-19 wave, as well as its diagnostics proceeding, and to discuss the challenges met in the time frame from the suspected diagnosis to the therapeutic decision in such patients. We conducted a retrospective multicentre case series study at six centres of Sudhalkar and Raghudeep group of hospitals in India. ROCM was confirmed in 38 (2.5%) of the 1546 patients admitted with SARS-CoV-2 infection. The average time to establish a diagnosis was 16 days. In total, 19 (50%) patients suffered from type 2 diabetes and were mostly treated with hypoglycaemic agents (in 90% of cases). The standard of care for SARS-CoV-2 management included systemic steroids therapy, intravenous remdesivir for 5 days, and concomitant prophylactic antibiotic therapy following admission. The median (IQR) blood glucose levels in all patients during the course of hospitalisation was 320 (250.5–375) mg/dl. A total of 16% of patients had an irreparable functional loss, and the mortality was 5%. We may hypothesise that excessive administration of antibiotics that profoundly affects human microbiota, coupled with poorly controlled glycaemia and unprotocolised haphazard steroid administration, contribute to a favourable setting for mucormycosis infections.

## 1. Introduction

Rhino-orbital-cerebral mucormycosis (ROCM) is a rare invasive fungal infection of the nasal and maxillary sinuses and the orbit. The causing agent, Mucorales, from the family of mucormycetes fungi, includes more than 300 species and may be responsible for opportunistic infections, especially in immunosuppressed patients [1]. Particular attention to the ROCM was brought about following the first and second epidemic COVID-19 waves in India and other countries [2,3], where mucormycosis was mediatised by several world-renowned media [4].

Acute SARS-CoV-2 infection, as well as the non-specific treatment (including steroids and monoclonal antibodies immune therapy), exposes COVID-19 patients to the risk of opportunistic infections, including mucormycosis. Several predisposing risk factors for the development of ROCM were identified retrospectively. These include uncontrolled diabetes, immunosuppression, hemochromatosis, ketoacidosis, and damage to the physical barriers of the immune system [5].

The diagnosis of ROCM is based on clinical signs and instrumental confirmation [6]. In the case of para-orbital spread of mucormycosis, several specific signs such as orbital cellulitis not responding to conventional treatment, orbital apex syndrome, ptosis, ophthalmoplegia, blurring vision or vision loss development, bring the patient or physician to consult an ophthalmologist. Unfortunately, the appearance of such symptoms often attests to an advanced stage of fungal invasion, with unfavourable functional (vision loss, eye removal) or even lethal (brain infection via the orbit fissures) outcomes [7]. The management of such patients is challenging, urging the need for systemic antifungal treatment combined with surgery [8,9].

We report here a case series of ROCM, managed by ophthalmologists, in six referring centres of Sudhalkar and Raghudeep group of hospitals in India. The purpose of our report is to describe the prevalence of ROCM in the context of SARS-CoV-2 infection during the second Indian COVID-19 wave, as well as its diagnostics proceeding, and to discuss the challenges met in the period from the suspected diagnosis to the therapeutic decision in such patients.

## 2. Materials and Methods

### 2.1. Study Design and Patient Selection

We conducted a retrospective multicentre, case series study at six centres of Sudhalkar and Raghudeep group of hospitals in India. Medical records of all adult patients admitted for COVID-19 Pneumonia from 1 February 2021 until 10 May 2021 were screened to identify those with suspected and confirmed ROCM. The ROCM diagnosis was based on the European Organisation for Research and Treatment of Cancer and the Mycoses Study Group Education and Research Consortium (EORTC/MSG) guidelines [10]. We analysed medical records of patients, alive at the time of ROCM diagnosis. The ethics committee of the Sudhalkar Eye Hospital, Vadodara, India, approved this study.

### 2.2. Data Collection

In patients with identified ROCM, the following data were collected: demographics, the signs and symptoms of COVID-19 infection, laboratory findings at admission and during the course of therapy, the treatment administered, the duration of hospitalisation, the duration of steroid therapy, the presenting complaints with particular reference to COVID-19 associated infections/sequelae, the therapy administered for COVID-19 associated complications, and final outcomes.

### 2.3. Objectives

The primary objective was the prevalence of ROCM in the studied population. The secondary objective included the analysis of patient characteristics and infection management.

### 2.4. Statistical Analysis

Results are expressed as frequency and percentage for categorical data, and as a median and interquartile range (IQR) for continuous variables. The chi-squared test was used, wherever appropriate. Statistical significance was set at *p* < 0.05.

## 3. Results

In the period from 1 February 2021 to 10 May 2021, 1546 patients were admitted with a COVID-19 pneumonia diagnosis. Of these, in 51 (3.2%) patients, the ROCM was suspected, and in 38 (2.5%) was confirmed. The median (IQR) follow-up for these patients was 52.5 (28–72) days.

### 3.1. Demographics and Systemic Comorbidities

The median (IQR) age was 54 years (32–74.5) and body mass index (BMI) was 24.5 (22.2–28.2), and 26 (68%) were males. In total, 15 (39%) patients had no comorbidities before the hospital admission, while 19 (50%) patients suffered from type 2 diabetes (median (IQR) HbA1c levels at admission 7.9 (6.7–9.1)). The presence of diabetes was associated with a higher risk to develop ROCM (OR: 2.89 95% CI [1.76–13], *p* = 0.023). Other comorbidities included hypertension in five patients (13%), hypothyroidism in four patients (10.5%), dyslipidaemia in three patients (8%), impaired renal function in one patient (2.6%), cardiac insufficiency in one patient (2.6%), and rheumatoid arthritis in one patient (2.6%).

In diabetic patients (*n* = 19), treatment included oral hypoglycaemic agents in 17 (90%) cases, meaning that only 2 (10%) patients had insulin therapy.

### 3.2. SARS-CoV2 Infection Management—Related Data

All patients admitted for management of COVID-19 pneumonia underwent a CT pulmonary scan, with the average high-resolution CT scan (HRC) score [11] at 13/25. In total, 25 (66%) patients required oxygen supplementation at admission, 7 (18%) patients required non-invasive ventilatory support initiated within 24 h of admission, and 6 (16%) patients required invasive ventilation. None of the patients had any clinical evidence of ROCM/secondary infection at admission. The median (IQR) duration of oxygen therapy (regardless of delivery system) prior to the development of symptoms suggestive of ROCM was 15.2 (10.5–21) days.

The standard of care included systemic steroids therapy, intravenous remdesivir for 5 days, and concomitant prophylactic antibiotic therapy following admission for all patients. As a primary steroid therapy, 14 (37%) patients received high-dose dexamethasone (16 mg daily) for 10 days, 19 (50%) patients received methylprednisolone (125 mg daily) for 7 days, and 5 (13%) patients received oral prednisolone in an average dose of 1 mg/kg/day for 14 days. All patients received tapering doses of oral prednisolone during 3 weeks following discharge. Multiple antimicrobial (beta-lactams, tetracyclines, and macrolides) and antiparasite (ivermectin) agents were administrated to all patients for the period from 3 to 10 days. Eight (21%) patients received oral voriconazole for 5 days for presumed invasive pulmonary aspergillosis. In addition, six (16%) patients developed antibiotic-induced diarrhoea and were managed successfully with oral metronidazole. Low-molecular-weight heparin was given in 27 (71%) patients for the entire duration of their hospitalisation. All patients received oral supplements (such as zinc and vitamin C).

### 3.3. Blood Glucose Levels

All 38 patients received insulin therapy during hospitalisation. The median (IQR) blood glucose levels in all these patients during hospitalisation was 320 (250.5–375) mg/dl over a median (IQR) time of 11 (7–14) days. Only 16 (42%) patients achieved blood glucose levels < 200 mg/dl at any point in time. Ketoacidosis occurred in two (5%) patients in a median time of eight days from corticoid therapy initiation.

### 3.4. Time to Onset of Mucormycosis and Ophthalmologic Symptoms

The median (IQR) time to diagnosis of ROCM from the day of hospital admission was 17.5 (12–22) days. A diagnosis of ROCM was established in 10 (26%) patients during hospitalisation, in 8 (21%) within a week of discharge, in 15 (39.5%) within the first two weeks of discharge, and in 5 (13%) within the first month after discharge. None of the patients developed any other form of mucormycosis (such as cutaneous/pulmonary). The median (IQR) hospitalisation time in these 38 patients was 23 (15–28) days.

Ocular involvement occurred in 21 (55%) patients. The main ophthalmic symptoms were ptosis in 19 patients (90%), ophthalmoplegia in 18 patients (47%), total vision loss in 15 patients (39%), and orbital oedema in 11 patients (29%). None of the patients had bilateral orbital involvement.

### 3.5. Histopathology and Culture Results

In total, 33 (86.8%) patients underwent surgical intervention for ROCM. Fungal elements were detected in all 33 patients, using the potassium hydroxide (KOH) wet mount preparation. Histologic findings confirmed the presence of perivascular fungal hyphae, along with the classic signs of tissue infarction and necrosis. The identified fungal hyphae were pauciseptate, with broad and right-angle branching, consistent with the clinical diagnosis. Their structure was confirmed with the periodic acid–Schiff (PAS) staining. Culture and species identification were carried out in 23 patients (69.7%). The most commonly identified species were Rhizopus (60.9%), followed by Mucor (21.7%) and Absidia (17.4%). As all specimens were obtained from sterile sites, the fungal growth in culture media, together with histopathological evidence of vascular necrosis and the presence of fungal hyphae in surgical samples, was conclusive for mucormycosis (proven invasive fungal disease according to EORTC/MSG consensus definition [10]).

Five patients (13.2%) who improved with medical management alone had possible invasive mucormycosis based on the EORTC/MSG consensus definitions for invasive fungal disease: the presence of one host factor (uncontrolled blood glucose levels/immunosuppression), clinical evidence, and radiologic evidence based on CT scan of the head and sinus region.

### 3.6. Therapy for Mucormycosis

All patients with established ROCM diagnosis were treated with intravenous amphotericin B (deoxycholate-23, liposomal-15) with a median (IQR) dose of 0.9 (0.3–1.0) mg/kg/day over a median (IQR) duration of 21.5 days (14–28). A total of 17 (45%) patients received additional therapy with oral posaconazole, with a loading dose of 600 mg on day 1, followed by a maintenance dose of 300 mg/day over a median (IQR) duration of 60 (42–80) days.

**Figure 1:**
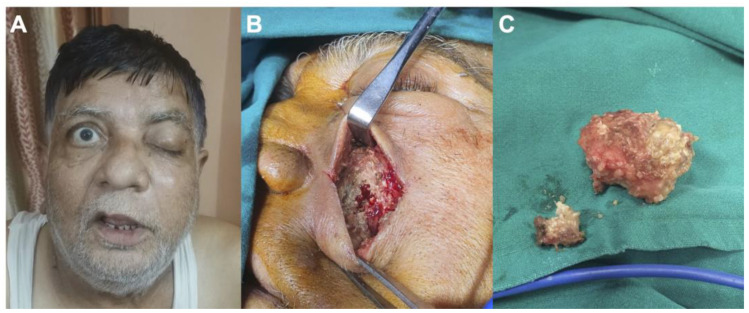
Case of a 65-year-old man suffering from mucormycosis following therapies for COVID-19 pneumoniae: (**A**) preoperative photography showing left ptosis and facial swelling; (**B**) preoperative photography demonstrating invasion of the sinus by mucormycosis; (**C**) preoperative photography showing surgical removal of the mucormycosis.

In total, 27 (71%) patients benefited from at least endoscopic debridement, 6 (16%) received orbital exenteration (eye loss), and 3 (8%) patients required hemi-mandibulectomy (Figure 1). Finally, five (13%) patients could be managed with medical therapy alone. Lethal outcomes were observed in two (5%) patients.

Patients with pre-existing diabetes were more likely to require aggressive interventions such as exenteration or hemi-mandibulectomy (OR: 4.42, 95% CI [3.03–5.27], *p* = 0.03). However, no association of lethal outcome and diabetes status was found.

## 4. Discussion

We described the ophthalmologist’s experience in the management of COVID-19 associated ROCM patients in six Indian hospital centres during the second pandemic wave. In our study, the prevalence of ROCM was 2.5%, and the average time to establish a diagnosis was 16 days. Only a quarter of patients (26%) benefitted from the in-hospital diagnostics of ROCM, whereas most of the patients were returning to the hospital for ophthalmologist consultation after discharge. The majority of patients had already established risk factors for mucormycosis and were exposed to well-known risk factors for ROCM. Finally, 16% of patients had an irreparable functional loss, and the mortality was 5%.

The immunosuppression (induced by SARS-CoV-2 infection and by steroid use), and exposure to high blood glucose levels (due to steroids use and preexisting diabetes) are well-depicted risk factors for any invasive fungal infection, including mucormycosis [12,13,14,15,16,17]. Steroid therapy was heterogeneous in our series, but it reflects the complexity of COVID-19 systemic therapy [18]. The in-hospital blood glucose level management was not optimal in the majority of our patients, and it is unknown whether the glucose control in these patients was successful at home. A clear association of unsuccessful glucose control and various infectious complications was already demonstrated in a large number of medical and surgical conditions.

The particularity of the fulminant mucormycosis is in relatively few general symptoms and predominant localisation in the oro-facial zone. This localisation puts patients at risk of severe functional loss—namely, loss of vision or even death. The context of the COVID-19 pandemic has led to a dramatic increase in the number of patients at risk and, therefore, the raise of ROCM. In our observation, 39% of patients had no comorbidities before hospitalisation. However, we did not consider these patients being free of any comorbidities before the COVID-19, because of the absence of precise medical history prior to the index hospitalisation. Previously published studies already reported a high incidence of ROCM in young patients with few comorbidities admitted for COVID-19 pneumonia, particularly in the western Indian state of Gujarat [12,13,14,19,20,21,22,23].

In our case series, patients with diabetes were at higher risk for extensive surgical interventions. Aggressive surgical debridement, combined with systemic antifungal therapy, probably accounted for a lower mortality rate during our observation. However, lethal outcomes associated with ROCM recurrence may have occurred later in the course of the disease.

One of the features of therapeutic interventions for COVID-19 pneumonia, particularly in India, is the wide administration of antibiotics to prevent secondary bacterial infection, even in immunocompetent patients. A veritable cocktail of antibiotics, ivermectin, and even voriconazole was administered in some patients preventively. The potential association of mucormycosis with the administration of voriconazole has been documented earlier [24]. We may hypothesise that such excessive administration of antibiotics profoundly affects human microbiota lining mucous membranes. The loss of protective function of microflora may contribute to the fungal invasion. Coupled with poorly controlled glycaemia and unprotocolised steroid administration, this establishes a more than favourable setting for mucormycosis infections.

The standards of hospital hygiene in India were already not optimal before the COVID-19 pandemic [25]. During the pandemic wave, the risk of environmental exposure in the hospital was undoubtedly multiplied. COVID-19 patients with weakened immune systems may have been exposed to different infectious pathogens, including fungi, through hospital air, surfaces, and potentially contaminated medical devices for oxygen delivery and respiratory support. Moreover, public health issues, particularly present during pandemics, such as domestic waste management, pose serious problems in India [26]. We may expect the continuous risk of exposure to Mucorales for immunocompromised post-COVID-19 patients in domestic settings, especially in rural areas. Therefore, the cases with onset of ROCM at 1 and 2 weeks post-discharge may be considered both as nosocomial (more probable) or community-acquired.

The small number of included participants and the retrospective nature of the study are the major limitations of our analysis. Different causative factors have to be studied; however, the heterogeneity in therapeutic approaches and the lack of medical history documentation prior hospitalisation precluded the use of advanced statistical methods such as propensity score matching. A large cohort analysis may be useful in this case. However, the descriptive nature of our cases may add to the literature allowing in the future a meta-analytic approach.

## 5. Conclusions

To conclude, 2.5% of adult patients hospitalised with SARS-CoV-2 infection suffered from ROCM, an uncommon but dangerous complication of COVID-19, leading to vision loss in 16% of cases and mortality in 5%. Aside from the effort to control known risk factors, physician awareness should be reinforced to enable earlier diagnostics and access to treatment. Further epidemiological studies are needed to improve the quality of management of such patients.

## Data Availability

All data are available upon request to the corresponding author.

## References

[1] Ak A.K., Gupta V. (2022). Rhino-Orbital Cerebral Mucormycosis. StatPearls.

[2] Sen M., Honavar S.G., Bansal R., Sengupta S., Rao R., Kim U., Sharma M., Sachdev M., Grover A.K., Surve A. (2021). Epidemiology, Clinical Profile, Management, and Outcome of COVID-19-Associated Rhino-Orbital-Cerebral Mucormycosis in 2826 Patients in India—Collaborative OPAI-IJO Study on Mucormycosis in COVID-19 (COSMIC), Report 1. Indian J. Ophthalmol..

[3] Fazeli M.A., Rezaei L., Javadirad E., Iranfar K., Khosravi A., Saman J.A., Poursabbagh P., Ghadami M.R., Parandin M.M., Dehghani A. (2021). Increased incidence of rhino-orbital mucormycosis in an educational therapeutic hospital during the COVID-19 pandemic in western Iran: An observational study. Mycoses.

[4] Dyer O. (2021). COVID-19: India sees record deaths as “black fungus” spreads fear. BMJ.

[5] Aranjani J.M., Manuel A., Razack H.I.A., Mathew S.T. (2021). COVID-19–associated mucormycosis: Evidence-based critical review of an emerging infection burden during the pandemic’s second wave in India. PLoS Neglected Trop. Dis..

[6] Yasmin F., Najeeb H., Naeem A., Dapke K., Phadke R., Asghar M.S., Shah S.M.I., De Berardis D., Ullah I. (2021). COVID-19 Associated Mucormycosis: A Systematic Review from Diagnostic Challenges to Management. Diseases.

[7] Tooley A.A., Bradley E.A., Woog J.J. (2022). Rhino-Orbital-Cerebral Mucormycosis—Another Deadly Complication of COVID-19 Infection. JAMA Ophthalmol..

[8] Kaur K., Gurnani B. (2021). Rhino-Orbital-Cerebral Mucormycosis in COVID-19 Patients—Taming the Black Evil with Pharmacological Weapons. Indian J. Pharmacol..

[9] Kothari N., Goyal A., Sharma A., Goyal S., Bhatia P.K., Yadav S. (2021). Mucormycosis: A Case Series of Patients Admitted in Non-COVID-19 Intensive Care Unit of a Tertiary Care Center during the Second Wave. Indian J. Crit. Care Med..

[10] Donnelly J.P., Chen S.C., Kauffman C.A., Steinbach W.J., Baddley J.W., Verweij P.E., Clancy C.J., Wingard J.R., Lockhart S.R., Groll A.H. (2020). Revision and Update of the Consensus Definitions of Invasive Fungal Disease From the European Organization for Research and Treatment of Cancer and the Mycoses Study Group Education and Research Consortium. Clin. Infect. Dis..

[11] Francone M., Iafrate F., Masci G.M., Coco S., Cilia F., Manganaro L., Panebianco V., Andreoli C., Colaiacomo M.C., Zingaropoli M.A. (2020). Chest CT score in COVID-19 patients: Correlation with disease severity and short-term prognosis. Eur. Radiol..

[12] Honavar S.G., Sen M., Lahane S., Lahane T.P., Parekh R. (2021). Mucor in a Viral Land: A Tale of Two Pathogens. Indian J. Ophthalmol..

[13] John T., Jacob C., Kontoyiannis D. (2021). When Uncontrolled Diabetes Mellitus and Severe COVID-19 Converge: The Perfect Storm for Mucormycosis. J. Fungi.

[14] Revannavar S.M., Supriya P.S., Samaga L., Vineeth V.K. (2021). COVID-19 triggering mucormycosis in a susceptible patient: A new phenomenon in the developing world?. BMJ Case Rep..

[15] Sharma S., Grover M., Bhargava S., Samdani S., Kataria T. (2021). Post coronavirus disease mucormycosis: A deadly addition to the pandemic spectrum. J. Laryngol. Otol..

[16] Prakash H., Chakrabarti A. (2021). Epidemiology of Mucormycosis in India. Microorganisms.

[17] Khatri A., Chang K.-M., Berlinrut I., Wallach F. (2021). Mucormycosis after Coronavirus disease 2019 infection in a heart transplant recipient—Case report and review of literature. J. Mycol. Med..

[18] Villar J., Añón J.M., Ferrando C., Aguilar G., Muñoz T., Ferreres J., Ambrós A., Aldecoa C., Suárez-Sipmann F., Thorpe K.E. (2020). Efficacy of dexamethasone treatment for patients with the acute respiratory distress syndrome caused by COVID-19: Study protocol for a randomized controlled superiority trial. Trials.

[19] Lancet T. (2021). India’s COVID-19 emergency. Lancet.

[20] Garg D., Muthu V., Sehgal I.S., Ramachandran R., Kaur H., Bhalla A., Puri G.D., Chakrabarti A., Agarwal R. (2021). Coronavirus Disease (COVID-19) Associated Mucormycosis (CAM): Case Report and Systematic Review of Literature. Mycopathologia.

[21] Dallalzadeh L.O., Ozzello D.J., Liu C.Y., Kikkawa D.O., Korn B.S. (2021). Secondary infection with rhino-orbital cerebral mucormycosis associated with COVID-19. Orbit.

[22] Mehta S., Pandey A. (2020). Rhino-Orbital Mucormycosis Associated With COVID-19. Cureus.

[23] Patel A., Kaur H., Xess I., Michael J., Savio J., Rudramurthy S., Singh R., Shastri P., Umabala P., Sardana R. (2019). A multicentre observational study on the epidemiology, risk factors, management and outcomes of mucormycosis in India. Clin. Microbiol. Infect..

[24] Pongas G., Lewis R., Samonis G., Kontoyiannis D. (2009). Voriconazole-associated zygomycosis: A significant consequence of evolving antifungal prophylaxis and immunosuppression practices?. Clin. Microbiol. Infect..

[25] Joshi S.C., Diwan V., Joshi R., Sharma M., Pathak A., Shah H., Tamhankar A.J., Lundborg C.S. (2018). “How Can the Patients Remain Safe, If We Are Not Safe and Protected from the Infections”? A Qualitative Exploration among Health-Care Workers about Challenges of Maintaining Hospital Cleanliness in a Resource Limited Tertiary Setting in Rural India. Int. J. Environ. Res. Public Health.

[26] Kothari R., Sahab S., Singh H.M., Singh R.P., Singh B., Pathania D., Singh A., Yadav S., Allen T., Singh S. (2021). COVID-19 and waste management in Indian scenario: Challenges and possible solutions. Environ. Sci. Pollut. Res. Int..

